# Daily Dosing of Rifapentine Cures Tuberculosis in Three Months or Less in the Murine Model

**DOI:** 10.1371/journal.pmed.0040344

**Published:** 2007-12-18

**Authors:** Ian M Rosenthal, Ming Zhang, Kathy N Williams, Charles A Peloquin, Sandeep Tyagi, Andrew A Vernon, William R Bishai, Richard E Chaisson, Jacques H Grosset, Eric L Nuermberger

**Affiliations:** 1 Center for Tuberculosis Research, Department of Medicine, Johns Hopkins University School of Medicine, Baltimore, Maryland, United States of America; 2 Department of International Health, Johns Hopkins Bloomberg School of Public Health, Baltimore, Maryland, United States of America; 3 Infectious Diseases Pharmacokinetics Laboratory, National Jewish Medical and Research Center, Denver, Colorado, United States of America; 4 Division of Tuberculosis Elimination, Centers for Disease Control and Prevention, Atlanta, Georgia, United States of America; Harvard School of Public Health, United States of America

## Abstract

**Background:**

Availability of an ultra-short-course drug regimen capable of curing patients with tuberculosis in 2 to 3 mo would significantly improve global control efforts. Because immediate prospects for novel treatment-shortening drugs remain uncertain, we examined whether better use of existing drugs could shorten the duration of treatment. Rifapentine is a long-lived rifamycin derivative currently recommended only in once-weekly continuation-phase regimens. Moxifloxacin is an 8-methoxyfluoroquinolone currently used in second-line regimens.

**Methods and Findings:**

Using a well-established mouse model with a high bacterial burden and human-equivalent drug dosing, we compared the efficacy of rifapentine- and moxifloxacin-containing regimens with that of the standard daily short-course regimen based on rifampin, isoniazid, and pyrazinamide. Bactericidal activity was assessed by lung colony-forming unit counts, and sterilizing activity was assessed by the proportion of mice with culture-positive relapse after 2, 3, 4, and 6 mo of treatment. Here, we demonstrate that replacing rifampin with rifapentine and isoniazid with moxifloxacin dramatically increased the activity of the standard daily regimen. After just 2 mo of treatment, mice receiving rifapentine- and moxifloxacin-containing regimens were found to have negative lung cultures, while those given the standard regimen still harbored 3.17 log_10_ colony-forming units in the lungs (*p* < 0.01). No relapse was observed after just 3 mo of treatment with daily and thrice-weekly administered rifapentine- and moxifloxacin-containing regimens, whereas the standard daily regimen required 6 mo to prevent relapse in all mice.

**Conclusions:**

Rifapentine should no longer be viewed solely as a rifamycin for once-weekly administration. Our results suggest that treatment regimens based on daily and thrice-weekly administration of rifapentine and moxifloxacin may permit shortening the current 6 mo duration of treatment to 3 mo or less. Such regimens warrant urgent clinical investigation.

## Abstract 2

Eric Nuermberger and colleagues found that after two months of treatment, mice with lung cultures positive for tuberculosis that received daily doses of rifapentine- and moxifloxacin-containing regimens converted to negative lung cultures. This finding could make possible the development of shorter treatment regimens for humans.

## Editors' Summary

**Background.:**

Every year, nearly 9 million people develop tuberculosis—a bacterial infection most commonly of the lungs—and about 2 million people die from the disease. Tuberculosis is caused by *Mycobacterium tuberculosis,* bacteria that are spread in airborne droplets when people with active tuberculosis sneeze or cough. Most infected people never become ill—their immune system successfully contains the infection. However, the bacteria remain dormant within the body and can cause disease years later if host immunity declines. Active tuberculosis can be cured by taking several antibiotics daily (for tuberculosis treatments, daily may mean five or seven times a week) for at least 6 mo. Combinations of drugs are needed to prevent the bacteria from developing resistance to the treatment, but also because of the complex biology of M. tuberculosis. During active tuberculosis, there are rapidly multiplying bacteria in the lungs but also less rapidly multiplying and near-dormant bacteria elsewhere in the body. Effective treatments contain a “bactericidal” drug such as isoniazid to kill the actively multiplying bacteria, a drug to kill the less actively multiplying bugs (for example, pyrazinamide), and a sterilizing drug (the most potent of which is rifampin) to kill the near-dormant bacteria and thus prevent the disease from recurring.

**Why Was This Study Done?:**

Unfortunately, many patients fail to complete this treatment because it is long and complicated and because the drugs may have unpleasant side effects. Poor adherence to treatment contributes to the emergence of drug resistance and means that people stay infectious for longer and are more likely to have relapses. Consequently, it is hampering global efforts to control tuberculosis. A shorter course of treatment might improve matters, but many researchers believe that this will require the development of new drugs and, although there are several promising candidates, it will be several years before they can be used in patients. In this study, therefore, the researchers asked whether better use of existing drugs could shorten treatment times. In particular, they studied tuberculosis in animals to investigate whether a long-lived rifampin-like drug called rifapentine combined with moxifloxacin (an alternative to isoniazid) might shorten treatment times.

**What Did the Researchers Do and Find?:**

The researchers used several different courses (“regimens”) of treatment containing rifapentine, moxifloxacin, and pyrazinamide, and the standard daily short-course regimen containing rifampin, isoniazid, and pyrazinamide to treat mice infected with M. tuberculosis. For each regimen, they measured its bactericidal activity by counting how many bacterial colonies could be grown from the lungs of the mice at specific times during the treatment, and its sterilizing activity by assessing the proportion of mice with any live bacteria in their lungs (a culture-positive relapse) after treatment completion. After 2 mo of treatment, the mice receiving the rifapentine- and moxifloxacin-containing regimens had negative lung cultures, a point not reached with the standard regimen until after 4 mo of treatment. Three months of treatment with daily or thrice-weekly rifapentine- and moxifloxacin-containing regimens was sufficient to prevent any culture-positive relapses, whereas the standard daily regimen had to be continued for 6 mo to achieve cure. Testing of additional drug combinations revealed that rifapentine is the most important drug in the new regimen and that simply replacing rifampin with rifapentine and retaining isoniazid also is sufficient to shorten the duration of therapy to 3 mo in this experimental model.

**What Do These Findings Mean?:**

These findings provide the first evidence that replacing rifampin with rifapentine might halve the length of therapy needed to cure tuberculosis. They also indicate that it might be possible to give the drugs thrice-weekly rather than daily as in the current therapy. The World Health Organization recommends that all tuberculosis treatment is supervised (so-called directly observed therapy) to ensure treatment adherence, so a regimen that requires only three doses a week for 3 mo would greatly reduce the resources needed to treat tuberculosis as well as potentially improving treatment adherence. However, it should be emphasized that the current study is experimental, and there may be important differences between how mice and people respond to the proposed drug regimens, both in terms of cure rates and side effects. Nevertheless, these results strongly suggest that the safety, tolerability, and efficacy of tuberculosis treatment regimens containing rifapentine and pyrazinamide, combined with either moxifloxacin or isoniazid, should be evaluated in people as soon as possible.

**Additional Information.:**

Please access these Web sites via the online version of this summary at http://dx.doi.org/10.1371/journal.pmed.0040344.

- The MedlinePlus encyclopedia contains a page on tuberculosis (in English and Spanish)
- The US National Institute of Allergy and Infectious Diseases provides information on all aspects of tuberculosis
- The US Centers for Disease Control and Prevention provide several fact sheets and other information resources about tuberculosis, including information for patients and caregivers about treatment adherence
- The World Health Organization provides a 2007 report on global tuberculosis control (in English with key findings in French and Spanish), information on the Stop TB initiative, and a recent bulletin on tuberculosis treatment (in English with an abstract in French, Spanish, and Arabic)

## Introduction

The development of simplified treatment regimens is a major priority of the Global Plan to Stop TB [1]. Novel regimens that are substantially shorter than the current 6-mo regimen are expected to simplify tuberculosis (TB) treatment, facilitate global implementation of directly observed therapy, improve treatment completion rates, and limit the emergence of multidrug-resistant TB.

It is widely believed that shortening the duration of TB therapy will require the development of new drugs with novel mechanisms of action [2]. Indeed, there is great optimism over several novel drug candidates that have recently entered Phase I and Phase II testing exclusively for the treatment of TB [3]. However, the immediate prospects for inclusion of these candidates into a new regimen remain uncertain. For example, TMC-207 has been highly regarded as a candidate for shortening therapy [4], but is currently being developed as a drug for multidrug-resistant TB rather than for drug-susceptible disease, at least in part due to a drug–drug interaction with rifampin. One recent analysis predicted the likelihood of introducing at least one successful anti-TB drug by 2010 to be <5% and the likelihood of introducing a novel regimen with at least two new drugs to be <1% [5]. Therefore, it is critical that existing drugs should be used in ways that optimize their potential activity.

Rifampin is recognized as the most important sterilizing drug in the modern short-course regimen. However, rifampin has a relatively short half-life of 2–4 h [6]. Rifampin-based regimens are less effective when the standard 600-mg daily dose is administered twice- or thrice-weekly as opposed to daily [7], and attempts to increase the rifampin dose to 1,200 mg or more in once- or twice-weekly regimens frequently result in an influenza-like syndrome [8–10]. Rifapentine is a cyclopentyl-substituted rifamycin which has been approved for use against TB in the United States since 1998 [11]. Because its half-life is five times longer than that of rifampin, it was developed as a rifamycin for once-weekly administration [12]. However, once-weekly treatment with rifapentine (600 mg) and isoniazid during the continuation phase of treatment, while effective, is inferior to twice- or thrice-weekly treatment with rifampin (600 mg) and isoniazid in patients at high risk of treatment failure and relapse, and current recommendations limit its use to HIV-seronegative persons with non-cavitary TB [11–14].

Low rifamycin drug exposure might explain the suboptimal efficacy of once-weekly rifapentine-containing regimens, particularly in the light of rifapentine's high protein binding [6,12,15,16]. On the supposition that the bactericidal activity of the rifamycins is correlated with the area under the serum concentration versus time curve divided by the minimum inhibitory concentration (AUC/MIC) [6,17,18], more frequent administration of rifapentine, with or without the use of higher doses, could greatly increase the rifamycin exposure over that obtained with 600 mg once per week and could result in substantially more potent treatment regimens (18). Rifapentine is well tolerated when administered once-weekly at doses of up to 1,200 mg and twice-weekly at doses of up to 600 mg [11,19,20]. The tolerability and safety of higher or more frequent doses have not been rigorously assessed. On the other hand, owing to concerns over the influenza-like syndrome and other adverse effects, there is reluctance to use rifampin at doses higher than 600 mg, especially with intermittent administration. In this context, we assessed the treatment-shortening potential of daily and intermittent treatment regimens with more frequent administration of rifapentine to significantly increase rifamycin exposure. Because we had previously demonstrated that the substitution of moxifloxacin for isoniazid substantially increases the bactericidal activity of rifamycin-based regimens [21,22], we also included this substitution in our test regimens.

## Methods

### Antimicrobials

Moxifloxacin (M) was kindly provided by Bayer Pharmaceuticals, isoniazid (H) and rifampin (R) were purchased from Sigma, and pyrazinamide (Z) was purchased from Fisher Scientific International. All drugs were either dissolved or suspended in water prior to oral gavage. Rifapentine (P) tablets, donated by Sanofi-Aventis, were ground into a fine powder, suspended in water, and briefly sonicated before gavage. Drug solutions were prepared weekly and stored at 4 °C.

### Aerosol Infection

Six-week-old BALB/c mice (Charles River) were aerosol infected with Mycobacterium tuberculosis H37Rv using the Middlebrook inhalation exposure system (Glas-Col) with a log-phase broth culture. All animal procedures were approved by the Johns Hopkins University Animal Care and Use Committee.

### Chemotherapy

**Figure 1:**
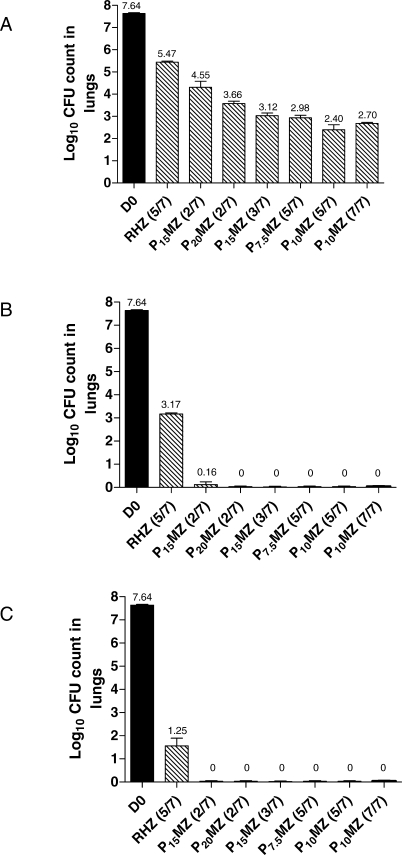
Lung Log_10_ CFU Counts in M. tuberculosis–Infected Mice CFU counts at treatment initiation (D0) and (A) after 1 mo, (B) after 2 mo, and (C) after 3 mo of treatment. Data are presented as means and standard error (error bars) (*n* = five mice per time point). H, isoniazid; M, moxifloxacin; Z, pyrazinamide; R, rifampin; P, rifapentine.

Treatment began 15 d after infection (on D0) in the first chemotherapeutic study (Table 1; Figure 1). Drugs were administered by oral gavage either 7 d/wk (7/7), 5 d/wk (5/7), 3 d/wk (3/7), or 2 d/wk (2/7) in the following doses: isoniazid (H), 25 mg/kg; rifampin (R), 10 mg/kg; pyrazinamide (Z), 150 mg/kg when administered (7/7) or (5/7), 225 mg/kg when administered (3/7), and 300 mg/kg when administered (2/7); moxifloxacin (M), 100 mg/kg twice per day; rifapentine, 7.5, 10, 15, and 20 mg/kg as indicated by the subscript numbers accompanying the abbreviation for the drug. Dosages for H, R, Z, and M were chosen to produce a serum AUC in mice equivalent to the AUC obtained with currently recommended human dosages [23,24]. For P, the 7.5-, 10-, 15-, and 20-mg/kg dosages are equivalent to 450-, 600-, 900-, and 1,200-mg dosages in humans, respectively [24].

**Table 1:**
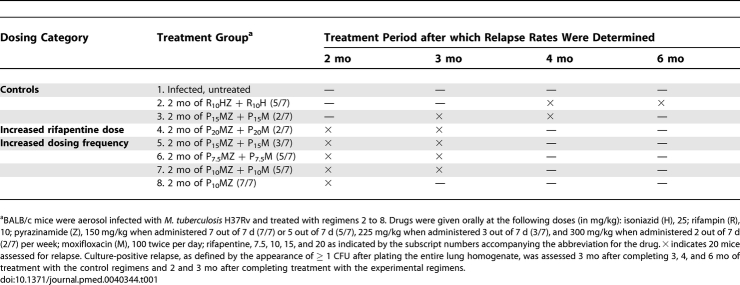
Scheme of Chemotherapy Experiment

At the start of treatment (D0), mice were block-randomized (by infection run) into eight groups (Table 1). Negative control mice went untreated, and positive control mice received standard daily (5/7) therapy (2 mo of rifampin plus isoniazid plus pyrazinamide [RHZ] followed by 2, 3, or 4 mo of RH) or 2 mo of twice-weekly P_15_MZ followed by 2 mo of twice-weekly P_15_M. To assess the impact of increasing the P dose, mice in Test Group 4 received 2 mo of P_20_MZ (2/7) followed by 1 mo of P_20_M (2/7). To assess the impact of increasing the frequency of drug administration to thrice-weekly, mice in Test Group 5 received 2 mo of P_15_MZ (3/7) followed by 1 mo of P_15_M (3/7). To assess the impact of increasing the frequency of drug administration to 5 d/wk, Test Groups 6 and 7 received 2 mo of rifapentine plus moxifloxacin plus pyrazinamide (PMZ) (5/7) followed by 1 mo of PM (5/7), with P administered at 7.5 and 10 mg/kg, respectively. Test Group 8 received true daily therapy with 2 mo of P_10_MZ (7/7).

**Figure 2:**
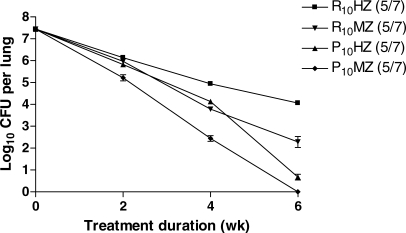
Bactericidal Activity of Daily (5/7) R_10_MZ, P_10_MZ, and P_10_HZ in Comparison to Standard Daily Therapy with R_10_HZ in M. tuberculosis–Infected Mice Treatment began when the bacillary burden reached 7.45 ± 0.03 log_10_ CFU per lung. After 10 wk of treatment and 3 mo of follow-up, the proportion of mice that were lung culture–positive was 100% and 53% for the R-containing regimens (R_10_HZ and R_10_MZ, respectively) and only 13% and 0% for the P-containing regimens (P_10_HZ and P_10_MZ, respectively) (data not shown in Figure). H, isoniazid; M, moxifloxacin; Z, pyrazinamide; R, rifampin; P, rifapentine.

In the second chemotherapeutic study (Figure 2), treatment began 14 d after infection (D0) with M. tuberculosis H37Rv. Mice were treated with daily (5/7) R_10_HZ, R_10_MZ, P_10_HZ, and P_10_MZ for 2, 4, 6, or 10 wk.

### Assessment of Treatment Efficacy

Treatment efficacy was assessed on the bases of lung colony-forming unit (CFU) counts and the proportion of mice with culture-positive relapse after treatment completion. To monitor the bacterial multiplication and establish baseline CFU counts prior to treatment, untreated mice were humanely killed the day after aerosol infection, with others killed 14 d later (D0), at the initiation of treatment. Subsequently, mice from all treatment groups were killed during treatment to assess bactericidal activity. Lungs were removed under aseptic conditions, placed in phosphate-buffered saline, homogenized, and plated on 7H11 agar enriched with 10% oleic acid-albumin-dextrose-catalase and supplemented with cycloheximide (10 μg/ml), carbenicillin (50 μg/ml), polymyxin B (25 μg/ml), and trimethoprim (20 μg/ml) to prevent contamination. Plates were incubated for 28 d at 37 °C in a 5% CO_2_ environment before CFU counts were determined.

The proportion of culture-positive mice was determined by humanely killing a cohort of mice 3 mo after the completion of treatment. Mice were considered to be culture-positive if ≥1 CFU was identified after plating the entire lung homogenate. In chemotherapy study 1 (Figure 1; Table 1), the proportion of mice with culture-positive relapse was determined after 2 and 3 mo of treatment for mice receiving the experimental regimens, and after 3, 4, and 6 mo for mice receiving the control regimens. In chemotherapy study 2 (Figure 2), the proportion of culture-positive mice was determined after 10 wk of treatment.

### Pharmacokinetic and Pharmacodynamic Analyses

Rifamycin pharmacokinetics were determined in uninfected female Swiss Webster mice after oral gavage with single doses of R_10_, P_7.5_, P_10_, P_15_, and P_20_. Drug concentrations in mouse plasma were determined by validated HPLC assays. Single-dose serum-concentration time curves were determined using non-compartmental techniques (WinNonLin Software). Steady-state pharmacokinetics assuming no autoinduction were simulated using nonparametric superposition, followed by simulation using a one-compartment model with first-order elimination. Parameter estimates (absorption rate constant, volume of distribution, and elimination rate constant) were adjusted to match the median single-dose serum-concentration time curve measured in mice, followed by simulation of various doses and frequencies of administration. Simulated pharmacokinetic parameter estimates were compared to actual published and unpublished steady-state concentrations determined for selected twice-daily and daily (5/7) regimens and showed excellent concordance [18]. Pharmacodynamic indices (e.g., free drug weekly AUC/MIC and maximum serum concentration [Cmax]/MIC ratios and time above MIC per week) were derived using the following MIC values: rifapentine, 0.06 μg/ml; rifampin, 0.25 μg/ml [25]. Free drug concentrations were estimated to be 2.5% and 17.5% of total serum concentrations for rifapentine and rifampin, respectively [6,11,17].

### Statistical Analyses

CFU counts were log_10_ transformed before analysis and expressed as CFU log_10_ ± standard error. Mean CFU counts were compared using one-way ANOVA followed by Dunnett's multiple comparison test (Stata 8.2). The proportions of mice relapsing after completing treatment were compared using Fisher's Exact test. Bonferroni's procedure was used to adjust the type I error rate for multiple comparisons.

## Results

### High-Dose Aerosol Infection with M. tuberculosis

In the first chemotherapeutic study, mice were aerosol infected with *M. tuberculosis* H37Rv in four separate runs. The day after infection (day 14), mice harbored 4.29 ± 0.04 tubercle bacilli per lung. Fourteen days later, at the start of treatment (D0), the mean lung log_10_ CFU counts increased to 7.73 ± 0.04, 7.56 ± 0.12, 7.62 ± 0.11, and 7.56 ± 0.04 (*p* = 0.12, one-way ANOVA) for mice infected during infection runs 1 to 4, respectively, indicating that all mice started treatment with an equal number of tubercle bacilli implanted in the lungs. All infected, untreated mice (*n* = 5) were humanely killed when they became moribund at 3 wk postinfection.

### Daily and Thrice-Weekly Rifapentine and Moxifloxacin-Based Therapy Is More Active than Standard Therapy

At completion of the first month of treatment, all intermittent and daily PMZ regimens had significantly greater bactericidal activity than the standard daily (5/7) RHZ regimen (Figure 1A). Compared to mice treated with RHZ (5/7), those treated with PMZ-based regimens had lung CFU counts that were between approximately 1 and 3 log_10_ lower than those of mice treated with RHZ (5/7) (*p* < 0.01, Dunnett's multiple comparison test), and the extent of bactericidal activity correlated with the frequency of drug administration (Figure 1A).

After 2 mo of treatment, all mice receiving a PMZ-based regimen had no detectable CFU, with the exception of two of the five mice treated with twice-weekly P_15_MZ, which harbored 1 and 5 CFU per lung, respectively (Figure 1B). By contrast, all mice treated with RHZ (5/7) remained culture positive with 3.17 log_10_ CFU per lung (*p* < 0.01, Dunnett's multiple comparison test). After 3 mo of treatment, all mice treated with a PMZ-based regimen remained culture-negative, while those treated with daily RHZ still harbored an average of 1.25 log_10_ CFU per lung (*p* < 0.01, Dunnett's multiple comparison test) (Figure 1C). All mice treated with daily RHZ were found to be culture negative after 4 mo of treatment, with the exception of one mouse which harbored 1 CFU per lung. Thus, mice receiving any intermittent or daily PMZ-based regimen other than P_15_MZ (2/7) achieved culture-negative conversion 2 mo earlier than those receiving RHZ (5/7).

To further investigate the activity of the P_10_MZ (5/7) regimen, we determined the individual contributions of P and M on the regimen's overall activity in a second chemotherapeutic study (Figure 2). The activity of the P_10_MZ (5/7) regimen was compared to that of the standard daily regimen of R_10_HZ (5/7), the well-characterized experimental regimen of R_10_MZ (5/7) [21,22], and P_10_HZ (5/7). After only 2 wk of therapy, treatment with P_10_MZ (5/7) was significantly more active than treatment with R_10_HZ, R_10_MZ, or P_10_HZ (*p* < 0.01, Dunnett's multiple comparison test), demonstrating the improved initial bactericidal activity obtained by substituting both P for R and M for H. After 6 wk of treatment with R_10_HZ, mice still harbored more than 4.0 log_10_ CFU per lung, whereas those treated with R_10_MZ and P_10_HZ harbored 2.28 log_10_ CFU and 0.68 log_10_ CFU per lung, respectively; mice treated with P_10_MZ were completely lung culture–negative. Therefore, in the standard regimen of R_10_HZ, the replacement of R_10_ with P_10_ results in a significantly greater increase in bactericidal activity than the replacement of H with M (*p* < 0.01, Dunnett's multiple comparison test).

### Rifapentine and Moxifloxacin-Based Therapy Produces Stable Cure More Rapidly than Standard Therapy

To assess the duration of treatment required to achieve stable cure, we determined the proportion of mice with culture-positive relapse after the end of treatment (Table 2). Although all PMZ regimens achieved lung culture conversion following 2 mo of treatment, there were considerable differences in the proportion of mice with culture-positive relapse at this time point (Table 2). In mice that received P_10_MZ (5/7) and P_10_MZ (7/7), only 35% and 20% relapsed, respectively, whereas 95% relapsed in groups receiving P_20_MZ twice-weekly or P_15_MZ thrice-weekly (*p* < 0.01, Fisher's Exact test). The proportion of mice with culture-positive relapse after treatment with daily P_7.5_MZ (5/7) was intermediate with 60% of mice relapsing. Three months of treatment with thrice-weekly or daily (5/7) regimens prevented relapse in all mice, with the exception of one of 20 mice treated with P_7.5_MZ (5/7). When PMZ was administered twice-weekly, 10%–20% of mice relapsed, but these differences were not significant when compared with the thrice-weekly and daily PMZ-based regimens (*p* = 0.110, Fisher's Exact test).

**Table 2:**
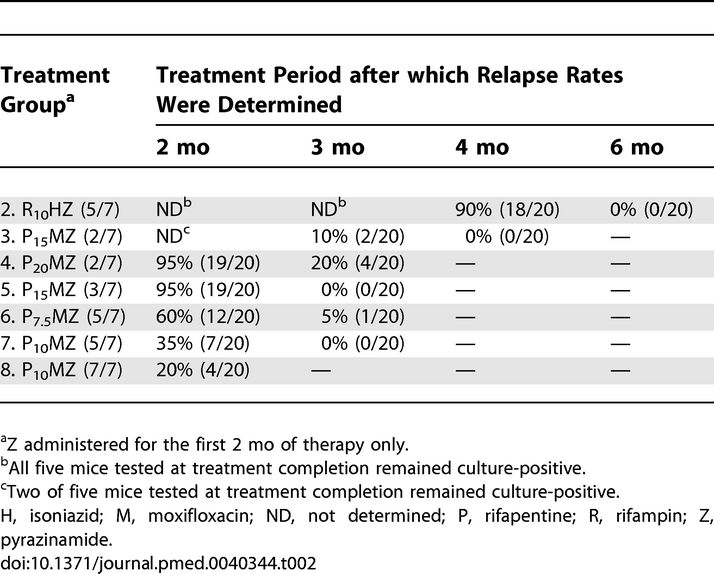
Culture-Positive Relapse Rates after Treatment with the Standard Daily Regimen (RHZ) and with Intermittent and Daily PMZ Regimens in M. tuberculosis–Infected Mice

Relapse was not assessed in mice treated with the standard control regimen, RHZ (5/7), until after 4 and 6 mo of treatment, where 90% and 0% of mice relapsed, respectively. Overall, these findings demonstrate that the duration of treatment required to achieve 0% relapse was decreased by at least 3 mo for the PMZ regimens administered daily or thrice-weekly as compared to the duration required with the standard daily regimen.

In the second chemotherapeutic study, we determined the proportion of mice with positive cultures 3 mo after completing 10 wk of treatment (Figure 2). Because lung cultures were not performed to document culture-negative conversion at the completion of treatment, we refrained from labeling this outcome as “relapse.” After 10 wk of treatment and 3 mo of follow-up, the proportion of mice that were lung culture–positive was 100% (15/15) and 53% (8/15) for the R-containing regimens (R_10_HZ and R_10_MZ, respectively) and only 13% (2/15) and 0% (0/15) for the P-containing regimens (P_10_HZ and P_10_MZ, respectively).

### Rifapentine-Based Regimens Provide Enhanced Rifamycin Exposure

**Figure 3:**
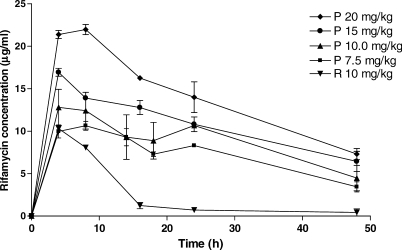
Mean Rifapentine and Rifampin Serum Concentrations versus Time Mice were administered a single dose of 7.5, 10, 15, and 20 mg/kg of rifapentine or 10 mg/kg of rifampin. P, rifapentine; R, rifampin.

**Figure 4:**
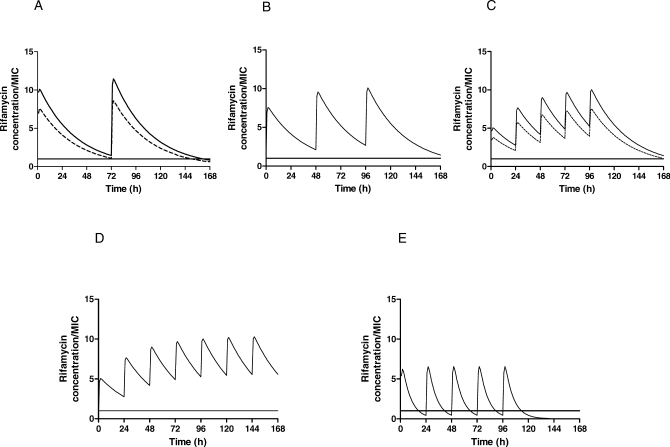
Steady-State Pharmacodynamic Simulations in Mice Treated with Rifapentine Compared with Rifampin Mice were treated with (A) twice-weekly (2/7) rifapentine 15 mg/kg (dashed line) and 20 mg/kg (solid line); (B) thrice-weekly (3/7) rifapentine 15 mg/kg; (C) daily (5/7) rifapentine 7.5 mg/kg (dashed line) and 10 mg/kg (solid line); (D) daily (7/7) rifapentine 10 mg/kg; and (e) daily (5/7) rifampin 10 mg/kg. Rifamycin concentrations are expressed in terms of free drug (2.5% and 17.5% free drug for rifapentine and rifampin, respectively). The free rifamycin concentration is below the MIC when the curve falls below the solid horizontal line. Pharmacodynamic parameter estimates are shown in Table 3.

Finally, in order to confirm the increased rifamycin exposure associated with P administration, we compared the single-dose pharmacokinetic parameters (Figure 3) and the simulated steady-state pharmacokinetic and pharmacodynamic parameters (Table 3) of intermittent and daily P administration, on the one hand, to those of daily R administration. The area under the serum concentration versus time curve (AUC_0–48__h_ in μg-h/ml) for total drug concentrations after a single dose of P was 341 ± 33, 417 ± 61, 503 ± 62, and 659 ± 73 for 7.5, 10, 15, and 20 mg/kg, respectively, compared to 116 ± 20 after a single dose of 10 mg/kg of R (*p* < 0.01, Dunnett's multiple comparison test). The weekly area under the serum concentration versus time curve (AUC_0–168 h_) for total drug concentrations at steady-state was between 2- and 4-fold higher for intermittent and daily P-based regimens compared to the daily R_10_ regimen (Table 3). After adjusting for differences in the minimum inhibitory concentration (MIC) and protein binding between P and R, the rifamycin exposure, as measured by both AUC_0–168 h_/MIC and the percentage of time above MIC per week for free drug, remained much higher in the P-based regimens (Table 3). Figure 4 depicts the simulated steady-state pharmacodynamic curves based on free drug concentrations for twice- and thrice-weekly, as well as daily (5/7, 7/7) P, compared to daily (5/7) R_10_.

**Table 3:**
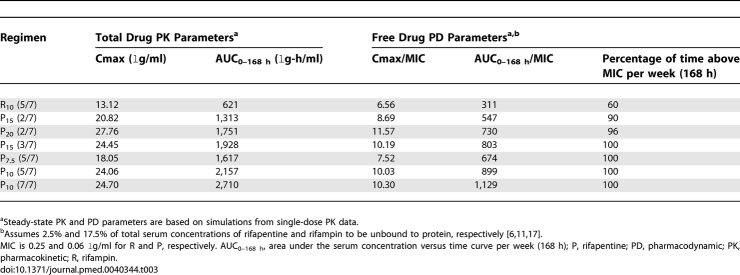
Steady-State Pharmacokinetic and Pharmacodynamic Parameters for Rifapentine and Rifampin in Mice

## Discussion

We set out to determine whether two currently registered drugs, rifapentine and moxifloxacin, could be used more effectively to improve the treatment of TB. Our results demonstrate that the combination of increasing rifamycin drug exposure by replacing rifampin with rifapentine and replacing isoniazid with moxifloxacin in the standard 6-mo daily treatment regimen may radically shorten the duration of therapy necessary to cure patients of TB. Treatment with P_7.5_MZ and P_10_MZ 5 d/wk (5/7) eliminated M. tuberculosis bacilli at a rate more than twice as fast as treatment with R_10_HZ (5/7). During the first month of treatment, the rates of kill were 1.17 and 1.31 log_10_ CFU/week for P_7.5_MZ and P_10_MZ (5/7), respectively, versus 0.54 log_10_ CFU/week for the standard control regimen (*p* < 0.01). All mice treated with PMZ (5/7) had negative lung cultures after 2 mo of treatment, while more than 4 mo was required to render all lung cultures negative in mice treated with RHZ (5/7). Finally, the proportion of mice relapsing after treatment with PMZ (5/7) regimens was 19 out of 40 (47.5%) and one out of 40 (2.5%) after 2 and 3 mo of treatment, respectively, compared to 18 out of 20 (90%) and 0 out of 20 (0%) after RHZ (5/7) for 4 and 6 mo, respectively. Relapse was not assessed after 5 mo of treatment with RHZ (5/7) in this study, but comparisons with other recent experiments in our model on the bases of the 4- and 6-mo relapse rates suggest that the 5-mo relapse rate would have been between 17% and 60% [18,24]. Therefore, daily (5/7) treatment with the PMZ-based regimens may permit the treatment duration to be reduced by at least 50%, from 6 mo to 3 mo, without sacrificing efficacy.

The long serum half-lives of P and M also might enable treatment-shortening regimens to be administered intermittently as opposed to daily, thus further reducing the resources necessary to administer supervised treatment [26]. To this end, decreasing the frequency of drug administration from 5 d/wk to 3 d/wk in our study did not significantly alter the potency of the PMZ regimen, provided the dose of P was increased from 7.5 mg/kg to 15 mg/kg. Administration of P_15_MZ thrice-weekly (3/7) produced a rate of kill similar to that of P_7.5_MZ (5/7) and prevented relapse in all mice after just 3 mo of treatment. Decreasing the frequency of drug administration to 2 d/wk resulted in reduced bactericidal activity compared to daily PMZ-based regimens. Still, the majority of mice receiving PMZ-based regimens twice-weekly (2/7) were cured after 3 mo of treatment, although 4 mo was required to prevent all relapses with the P_15_MZ (2/7) regimen. Thus, the pharmacokinetics of P and M give PMZ-based regimens the flexibility to be administered daily or intermittently while preserving some treatment-shortening potential.

The finding that all PMZ regimens were more active than the standard daily R_10_HZ regimen may be explained largely by the greater rifamycin exposure obtained by substituting P for R, as a consequence of the former's longer elimination half-life. In support of this premise, we found that the AUC_168 h_/MIC and the percentage of time above MIC were substantially greater for P-based regimens compared to the R-based regimen. Specifically, the free drug AUC_168 h_/MIC values ranged from 26% to 180% greater for P-based regimens, as compared to daily R (10 mg/kg), and free drug concentrations exceeded the MIC for at least 90% of the dosing interval in each P-based regimen, but for only 60% of the time in the daily R regimen (Table 3). A similar pharmacodynamic advantage may be expected in humans because rifamycin kinetics are similar between mice and humans [23,24]. It is intriguing that the P_7.5_MZ (5/7) regimen, which is much more potent than the control regimen, produced a rifamycin Cmax/MIC value similar to that of the control regimen; however the AUC_0–168 h_/MIC and the percentage of time above MIC values were significantly higher. If, as we surmise, the sterilizing activities of the rifamycins are linked to the AUC/MIC and/or the weekly time above MIC (or time above some other parameter), then one might rightfully question whether increasing the dose of R to a sufficient level could lead to similar improvements in sterilizing activity as seen here with P. While there is great reluctance to use higher doses of R in intermittent regimens owing to the past occurrence of serious adverse events [8–10], the use of higher daily doses of R deserves further evaluation. In the end, the best rifamycin for daily administration may be the one that is best tolerated at the highest exposure level. Further exploration of the pharmacodynamics of rifamycin sterilizing activity would be helpful in this endeavor by identifying the parameter that correlates best with such activity and the target exposures necessary for optimal activity. Evaluating the maximum tolerated dose in humans of both rifampin and rifapentine would then enable a rational choice of rifamycin and associated dose for further clinical development.

The substitution of M for H also contributes to the enhanced activity of PMZ regimens over RHZ, as demonstrated in our second chemotherapeutic study (Figure 2) and in our previous study of twice-weekly regimens [18]. However, the substitution of M for H does not contribute as much treatment-shortening potential as the substitution of P for R. This important finding suggests that regimens based solely on the substitution of P for R also are capable of significantly shortening the duration of treatment, perhaps to 3 mo or less. The margin of benefit obtained with substituting M for H in a P-based regimen may be small enough that PHZ regimens may prove more cost-effective than PMZ regimens in resource-limited settings.

Our results with daily and intermittent P-based regimens are especially encouraging in the light of recent clinical findings that intermittent treatment with RHZ is less active than the same treatment administered daily [27–29]. For example, a recent systematic review of published clinical trials using 6-mo rifamycin-based treatment regimens found that treatment with intermittent regimens, with the exception of a daily initial phase followed by a thrice-weekly continuation phase, was associated with a greater risk of relapse [27]. Abbreviated twice- and thrice-weekly P-based regimens may provide for highly simplified treatment regimens that are more potent than the standard daily rifampin-based regimen.

Several caveats must be considered when extrapolating these results from the murine model to the treatment of humans. First and foremost is the reliability with which the murine model predicts the outcomes of TB chemotherapy in humans. When care is taken to create a bacillary burden that is representative of that found in human cavitary TB and to administer drugs at human-equivalent doses, the mouse model has a strong track record of predicting the duration of treatment necessary to attain acceptable cure rates in human TB with various regimens, including RHZ [23]. More recently, results in the murine model correlated closely with the results of a Phase II study to investigate the effect of substituting M for ethambutol (E) in the standard four-drug regimen of RHZ plus E [21,30]. Results from additional Phase II and Phase III studies, some of which are recently concluded or underway, are expected to shed additional light on whether the substitution of M for E or H in the standard four-drug regimen has a beneficial effect, as first demonstrated in the murine model [22].

A second caveat is that the safety and tolerability of the PMZ regimens studied here have yet to be investigated in humans. P has been well tolerated when administered at 10 mg/kg twice-weekly or at 15 and 20 mg/kg once-weekly for 2 mo or more [11,19,20], but there are no published studies investigating daily or thrice-weekly administration of P or more frequent administration of 15- and 20-mg/kg doses. Thus far, daily administration of M in combination with first-line TB drugs for 2 mo appears to be safe and well tolerated [30]. Treatment for longer durations has also been well tolerated in anecdotal experiences [31–33].

In conclusion, we have used a well-established murine model to reevaluate the potential contribution of P to the treatment of TB. This study has identified novel regimens that are dramatically more effective than the standard short-course RHZ regimen. We believe their greater efficacy is due primarily to the greater rifamycin exposure obtained when P is administered more frequently than once-weekly. A more modest beneficial effect of replacing H with M was also observed. Because of the prolonged elimination half-life of P, both intermittent and daily P-based regimens may have the potential to shorten the duration of treatment, perhaps to 3 mo or less. These results underscore the value of understanding antimicrobial pharmacodynamics for new and existing TB drugs in order to maximize their potential contribution and support the urgent evaluation of the safety, tolerability, and efficacy of P-based regimens for the treatment of TB.

## Abbreviations

AUC/MIC: area under the serum concentration versus time curve divided by the minimum inhibitory concentration
CFU: colony-forming unit
Cmax: maximum serum concentration
E: ethambutol
H: isoniazid
M: moxifloxacin
MIC: minimum inhibitory concentration
P: rifapentine
PMZ: rifapentine plus moxifloxacin plus pyrazinamide
R: rifampin
RHZ: rifampin plus isoniazid plus pyrazinamide
TB: tuberculosis
Z: pyrazinamide
